# Determinants of catastrophic healthcare spending in Portugal (2005–2022)

**DOI:** 10.1016/j.hpopen.2026.100171

**Published:** 2026-04-15

**Authors:** Tzi Kieu Tao, Pedro Pita Barros

**Affiliations:** Nova School of Business and Economics, R. da Holanda 1, 2775-405 Carcavelos, Portugal

**Keywords:** Catastrophic Health Expenditure (CHE), Determinants, Healthcare services, Portugal

## Abstract

The study analyses the extent, evolution and impoverishing effects of catastrophic health expenditures (CHE) in Portugal from 2005 to 2022. The analysis also identifies factors associated with the occurrence of CHE. The results show a progressive reduction in financial burden with increased access to healthcare services. CHE incidence decreased from 9.42% (2005) to 5.04% (2022). Reduced access to the Portuguese NHS was associated to increased reliance on private services and out-of-pocket costs. Barriers, unmet needs and challenges regarding the protection of vulnerable groups remain, including the elderly and individuals incapacitated to work. Households with at least one senior show a particular vulnerability to CHE. Regional disparities are reinforced by underdeveloped healthcare infrastructure, limited access and financial barriers.

## Highlights

• CHE incidence in Portugal declined progressively from 9.42% (2005) to 5.04% (2022).
• Elderly households and those unable to work face the greatest CHE vulnerability.
• Income-related disparities persist, alongside notable geographic inequalities.
• Reduced NHS access led to greater reliance on private care and out-of-pocket costs.

## 1 Background

Illness is considered as one of the most unpredictable and significant economic shocks that households can experience [1]. Research has consistently demonstrated that illness can result in adverse financial outcomes through two main pathways: out-of-pocket payments (OOPs) and income losses stemming from decreased labor supply or productivity [2], [3].

Out-of-pocket payments refer to the direct expenses households incur at the point of care, typically using personal income or savings, without reimbursement from third-party payers such as insurance providers or government programs. These expenditures may include co-payments, fixed service fees, and other health-related charges [4].

Within health systems, OOPs, particularly those tied to cost-sharing mechanisms, are often employed to enhance efficiency by mitigating overutilization and addressing demand-side moral hazard [5]. The underlying logic suggests that when patients bear some of the financial responsibility for their care, they will make more judicious healthcare decisions, thereby reducing unnecessary utilization. However, while such mechanisms may help manage healthcare resource use, they can also lead to impoverishment, highlighting the trade-off between managing demand and risking financial hardship [6].

Understanding the full scope of out-of-pocket payments requires distinguishing between different types of healthcare expenditures. Policy-driven OOPs function as regulatory tools within formal insurance frameworks, designed to influence patient behavior and control system costs. In contrast, access-driven payments, such as expenditures for over-the-counter medications or privately financed consultations, occur outside insurance coverage and are typically driven by access barriers or individual preferences rather than cost-containment strategies.

Out-of-pocket health expenditures are classified as catastrophic when they exceed a specific percentage or threshold of a household’s resources [7]. Such expenditures can create financial barriers to accessing care, resulting in unmet medical needs and, in some cases, impoverishment [7].^1^ Moreover, when households must allocate large portions of their limited incomes to healthcare, they are often left with insufficient resources for essentials like food, shelter, and education [7], [8]. This financial strain can force households to borrow money or sell assets to cover healthcare costs [9].

The relationship between poverty and health is well-established, with individuals from lower-income groups typically experiencing worse health outcomes, including shorter life expectancies and a higher prevalence of chronic conditions [8], [10], [11]. When health deteriorates, productivity may decline, reducing household income and limiting the ability to invest in health and well-being [2]. This dynamic deepens socioeconomic disadvantage and reinforces health inequalities across income groups.

Providing financial protection against the cost of healthcare is one of the core objectives of health systems, as defined by the World Health Organization [12]. In line with these goals, the WHO [12] emphasizes that health system financing must guarantee universal access to healthcare services by providing adequate funding and creating appropriate incentives for providers. However, even in countries with universal coverage for essential services, variations in the scope of coverage and the degree of cost-sharing can lead to affordability challenges for certain segments of the population [13]. Data indicate that many high-income countries continue to rely significantly on out-of-pocket payments. In the WHO European Region, OOPs accounted for more than 20% of total health expenditure in 14 out of 34 high-income countries in 2022 [14]. Among OECD and EU countries, Portugal stands out with one of the highest shares of out-of-pocket payments in total health expenditure [15]. Since 2005, the share of out-of-pocket payments in health expenditure in Portugal has risen from 23.7% to 29.8% in 2023, while the OECD average declined from 23.2% to 19.5% [16], highlighting Portugal’s growing divergence in financing patterns. This ongoing issue emphasizes the need for detailed analysis and targeted policy responses to protect vulnerable populations.

Previous studies have estimated the incidence of CHE in Portugal and identified population groups that are particularly vulnerable. They have also shown that pharmaceuticals account for a large share of out-of-pocket payments. Existing evidence is largely based on limited timeframes or single survey waves. Moreover, earlier analyses often relied on CHE measures that may underestimate financial hardship among poorer households. The present study extends the existing literature by examining the incidence and impoverishing effects of catastrophic health expenditures (CHE) in Portugal, as well as the factors associated with their occurrence, using data from four waves of the Portuguese Household Budget Surveys conducted in 2005/2006, 2010/2011, 2015/2016, and 2022/2023. This timeframe allows for a comprehensive assessment of how CHE has evolved over nearly two decades, encompassing three major economic shocks, including the 2008 economic crisis, the public debt crisis and the COVID-19 pandemic. These events shaped healthcare and household finances and reflect significant policy reforms. In 2011, user charges were increased as part of fiscal consolidation measures introduced during the international bailout of Portuguese public finances, alongside an expansion of exemption categories aimed at protecting vulnerable groups. User charges were abolished for primary care services in 2021 and for diagnostic and laboratory services in 2022, while referrals to emergency services remained exempt from user charges throughout. In parallel, changes to pharmaceutical cost-sharing were also introduced during this period. These developments occurred in the context of increasing poverty and financial pressure on households. The COVID-19 pandemic further disrupted healthcare access and household finances, highlighting the relevance of analysing this timeframe [17], [18], [19].

This study adopts a framework published by the World Health Organization Regional Office for Europe. Previous CHE measures, although widely used in the literature, present several limitations that restrict their ability to capture financial hardship among the poorest households and may therefore lead to underestimation of the true burden. The approach adopted in this study provides a more granular assessment of financial hardship than has previously been applied in the Portuguese context. In addition, the analysis refines the assessment of financial vulnerability by introducing varying thresholds for defining basic needs and by examining differences across household groups, thereby offering insights into how household size and composition are associated with exposure to health-related financial risk.

By combining this methodological approach with an extended longitudinal perspective, this study provides new evidence on the evolution of CHE and financial protection in the Portuguese health system. The findings contribute to the broader debate on health system reforms aimed at strengthening financial protection and promoting equity.

Section 1 provides a brief overview of the concept and measurement of catastrophic health expenditure. Section 2 describes the data and methodology employed in the analysis. Section 3 presents the results, followed by Section 4, which discusses the limitations of the study. Finally, Section 5 concludes.

## 2 Catastrophic healthcare expenditure

The aim of measuring catastrophic health expenditures (CHE) is to assess the incidence of financial burden that households experience due to healthcare costs [20]. The incidence of CHE varies across countries and regions of the world, influenced by factors such as health financing mechanisms and economic development [20]. Wagstaff et al. [20] have found a positive correlation between CHE and both GDP per capita, and the share of GDP spent on health. Xu et al. [21] have reached similar conclusions in their earlier research, suggesting that while increased healthcare spending may improve access to services, it does not necessarily enhance financial protection. They proposed that factors such as greater service availability leads to increased use of expensive technologies, and higher healthcare prices which in turn contribute to higher CHE rates [21]. Moreover, Wagstaff et al. [20] reported that CHE is negatively correlated with the share of health expenditure channeled through social security funds and other government agencies, highlighting the importance of public health insurance in mitigating financial risk.

CHE has been defined and measured using various approaches. The notion that out-of-pocket (OOP) payments may have impoverishing side effects has been recognized for decades, with interest rising and falling over time [22], [23], but it has been gaining increasing attention from researchers since the early 2000s [6]. Earlier research has already explored the distinction between high-cost and catastrophic illnesses^2^, as well as the concept of ability to pay [23], [24]. Berki [25] defined catastrophic health expenditure as healthcare spending that consumes a substantial portion of a household's budget, thereby compromising the ability to maintain its standard of living. Similarly, Russell [24] emphasized the opportunity costs associated with such spending, highlighting trade-offs households face when allocating resources to healthcare at the expense of other essential needs. However, Wagstaff and van Doorslaer [26] formalized two key approaches to defining CHE. The first evaluates out-of-pocket (OOP) payments as a proportion of total expenditure, considering them catastrophic when they exceed thresholds ranging from 2.5% to 15% [26]. The second approach assesses OOP payments relative to a household's capacity to pay, defined as total expenditure minus subsistence needs, with thresholds set between 10% and 40% [26]. The capacity-to-pay approach differs from other methods by focusing only on the portion of a household's budget remaining after accounting for basic needs. This assumes that the remaining budget is entirely available for healthcare expenses, which distinguishes it from approaches that consider total income or expenditure without such adjustments [27]. Additionally, the authors developed measures to evaluate the incidence, intensity, distribution, and impoverishment effects of OOP payments [26], offering a comprehensive framework for analysing financial hardship caused by healthcare costs. Xu et al. [28] refined the definition of catastrophic health expenditures (CHE) and the conditions under which they occur. They first standardized the definition of CHE as occurring when out-of-pocket (OOP) payments constitute 40% or more of a household’s capacity to pay [28]. The authors identified three prerequisites for CHE: the presence of health services requiring OOP payments, low household capacity to pay, and the absence of prepayment mechanisms for financial protection, such as health insurance Xu et al. [28]. Additionally, they acknowledged limitations in CHE measures, particularly their inability to account for households so impoverished that they lack access to medical services altogether [28]. The methodology later developed by Xu et al. [29] has been widely recognized for its robustness in capturing the financial burden of healthcare costs and has since been adopted by the World Health Organization (WHO) as the standard for assessing financial protection and health system performance globally [30], [31], [32].

In Portugal, studies covering the period from 2000 to 2015 indicate a decline in the incidence of CHE, suggesting improved financial protection over the years [6], [33], [34]. Several factors were identified as determinants. Although income is a contributing factor with a relatively small direct economic impact, the highest prevalence of CHE is consistently observed among households in the poorest income quintile [6], [34]. Age is another important determinant, with older individuals more likely to experience CHE due to greater healthcare needs combined with limited financial resources [6], [34]. Household composition is also an important factor; households with older members are more likely to experience CHE, whereas those with children or larger families are less likely to be affected [6], [33], [34]. Lastly, households with a member or members unable to work are more susceptible to CHE [6].

## 3 Data and methods

### 3.1 Data

The data used in this study are drawn from the Portuguese Household Budget Surveys (PHBS) conducted in 2005/2006, 2010/2011, 2015/2016 and 2022/2023. These surveys utilize a stratified and clustered sampling design to collect information from a representative sample of households living in non-collective dwellings across the country [35], [36], [37], [38]. The series of household budget surveys in Portugal began in 1967/1968, with the PHBS formally introduced in 2005/2006 and subsequently carried out every five years by Statistics Portugal (INE) [39]. Sampling weights developed by INE are applied. For simplicity, these periods are referred to as 2005, 2010, and 2015, 2022 respectively. The four samples used in this analysis consist of 10,403; 9,489; 11,398 and 11,701 households, corresponding to each period in chronological order [35], [36], [37], [38]. The surveys were administered through in-person interviews conducted throughout the entire year to capture seasonal and temporal variations [39]. For the expenditure component, participants completed detailed tables documenting their purchases, costs, and related information over a two-week period [39]. The collected data includes household expenditures, covering areas such as food, clothing, furniture, kitchen appliances, electronics, utilities, insurance, leisure, and transportation, alongside information on household composition, income, education level, employment status, and income sources [39]. The variables used for the analysis are detailed in Tables 1 and 2.

**Table 1:** Variables.

Variable	Definition
Total Household Expenditure	Total Household expenditure excluding imputed rent.
Out-of-pocket Payments	Co-payments, fixed service fees, and other health-related charges in the following fields: Medicines and Pharmaceutical Specialities, Other Medical and Pharmaceutical Products, Therapeutic Appliances and Supplies, Medical Services, Dental Services, Diagnostic Aids, Nursing and Paramedical Services, Other Non-Hospital Services, Hospital Services. Out-of-pocket Payments do not include insurance premiums.
Capacity-to-pay	Household's Total Expenditure (not including imputed rent) minus a basic needs threshold. Negative Capacity-to-pay indicates that total expenditure is insufficient to meet basic needs.
Income (in 1000€)	Disposable annual income of the household, in Euros/1000.
Household head gender	Takes the value 0 if male and 1 if female.
Household head age	Age of the Household Head. 2005: Groups 1 to 14, from 0-14 years (Group 1), then every 5 years, to 75+ years (Group 14) 2010, 2015, 2022: Group 1 to 18, from 0-4 years (Group 1), then every 5 years, to 85+ years (Group 18)
Average Household Age	Arithmetic mean age of all household members, representing the household’s overall age composition.
Household Head completed education	Equal 1 if the household head (HH) has the respective level of education; 0 otherwise. HH with no education is the base group in econometric modelling. 4th, 6th, 9th year, 12th year (Secondary and post-secondary); Higher education
Household Size	Number of individuals per household.
Senior Ratio	Number of individuals above 65 / Number of household members
Junior Ratio	Number of individuals =<14 / Number of household members
Urban	Equal 1 if the household is situated in an urban area, 0 otherwise. Urban is the omitted variable in the econometric modelling.
Semi-urban	Equal 1 if the household is situated in a semi-urban area, 0 otherwise.
Rural	Equal 1 if the household is situated in a rural area, 0 otherwise
North, Center, Lisbon, Algarve, Azores, Madeira or Alentejo (omitted region in the econometric modelling)	NUTS II Regions; Equal 1 if the household is situated in the regions, 0 otherwise.
Employed ratio	Fraction of employed individuals per household.
Unemployed ratio	Fraction of unemployed individuals per household.
Retired	Fraction of retired individuals per household.
Incapacity to work	Fraction of individuals that are incapable of work per household.

**Table 2:** Descriptive statistics.

Characteristics				Mean	σ	Min	Max
Total Household Expenditure			2005	12,936.85	10,852.94	108.00	154,509.41
			2010	14,873.86	11,870.28	276.00	127,270.87
		
			2015	15,074.02	11,408.26	427.96	182,049.95
		
			2022	16,635.69	12,335.37	0.00	196,370.80
		
Out-of-pocket payments			2005	1,038.03	1,764.50	0.00	48,316.00
		
			2010	1,169.16	1,875.65	0.00	37,440.00
		
			2015	1,125.63	1,241.12	0.00	19,877.76
		
			2022	978.31	1,279.67	0.00	24,528.00
		
Capacity-to-pay			2005	9,479.25	10,413.96	−5,956.96	149,404.23
		
			2010	10,916.71	11,383.82	−12,065.52	125,536.73
		
			2015	10,684.64	11,012.37	−11,241.43	178,713.67
		
			2022	11,912.92	11,773.57	−12,140.78	189,728.78
		
Income (in 1000€)			2005	16.46	15.98	0.50	460.69
		
			2010	21.99	15.98	1.57	210.47
		
			2015	23.08	17.94	0.47	354.27
		
			2022	30.37	21.63	0.63	358.32
		
Household head gender			2005	0.31	0.46	0.00	1.00
		
			2010	0.36	0.48	0.00	1.00
		
			2015	0.42	0.49	0.00	1.00
		
			2022	0.42	0.49	0.00	1.00
		
Household head age			2005	9.36	3.03	2.00	14.00
		
			2010	11.23	3.20	4.00	16.00
		
			2015	11.67	3.21	4.00	18.00
		
			2022	11.87	3.29	4.00	18.00
		
Household Head completed education			2005	0.42	0.49	0.00	1.00
4th year							
			2010	0.36	0.48	0.00	1.00
		
			2015	0.29	0.45	0.00	1.00
		
			2022	0.37	0.48	0.00	1.00
		
6th year			2005	0.13	0.33	0.00	1.00
		
			2010	0.12	0.33	0.00	1.00
		
			2015	0.11	0.32	0.00	1.00
		
			2022	0.16	0.36	0.00	1.00
		
9th year*			2005	0.11	0.31	0.00	1.00
		
			2010	0.14	0.35	0.00	1.00
		
			2015	0.16	0.37	0.00	1.00
		
			2022	0.16	0.36	0.00	1.00
		
12th year			2005	0.08	0.27	0.00	1.00
(Secondary and post-secondary)							
			2010	0.11	0.31	0.00	1.00
		
			2015	0.14	0.35	0.00	1.00
		
			2022	0.18	0.38	0.00	1.00
		
Higher education			2005	0.07	0.25	0.00	1.00
		
			2010	0.12	0.32	0.00	1.00
		
			2015	0.18	0.38	0.00	1.00
		
			2022	0.24	0.43	0.00	1.00
		
Household Size			2005	2.73	1.35	1.00	13.00
		
			2010	2.57	1.26	1.00	12.00
		
			2015	2.55	1.24	1.00	16.00
		
			2022	2.42	1.18	1.00	10.00
		
Senior Ratio			2005	0.31	0.42	0.00	1.00
		
			2010	0.32	0.43	0.00	1.00
		
			2015	0.31	0.42	0.00	1.00
		
			2022	0.37	0.44	0.00	1.00
		
Junior Ratio			2005	0.10	0.18	0.00	1.00
		
			2010	0.08	0.17	0.00	1.00
		
			2015	0.10	0.18	0.00	1.00
		
			2022	0.07	0.16	0.00	1.00
		
Urban			2005	0.71	0.45	0.00	1.00
		
			2010	0.69	0.45	0.00	1.00
		
			2015	0.72	0.44	0.00	1.00
		
			2022	0.74	0.43	0.00	1.00
		
Semi-urban			2005	0.19	0.39	0.00	1.00
		
			2010	0.20	0.40	0.00	1.00
		
			2015	0.15	0.36	0.00	1.00
		
			2022	0.15	0.36	0.00	1.00
		
Rural			2005	0.18	0.38	0.00	1.00
		
			2010	0.19	0.40	0.00	1.00
		
			2015	0.17	0.37	0.00	1.00
		
			2022	0.16	0.37	0.00	1.00
		
North			2005	0.40	0.39	0.00	1.00
		
			2010	0.20	0.40	0.00	1.00
		
			2015	0.19	0.40	0.00	1.00
		
			2022	0.20	0.40	0.00	1.00
		
Center			2005	0.36	0.36	0.00	1.00
		
			2010	0.16	0.37	0.00	1.00
		
			2015	0.14	0.34	0.00	1.00
		
			2022	0.12	0.33	0.00	1.00
		
Lisboa			2005	0.33	0.33	0.00	1.00
		
			2010	0.15	0.36	0.00	1.00
		
			2015	0.22	0.42	0.00	1.00
		
			2022	0.23	0.42	0.00	1.00
		
Algarve			2005	0.36	0.36	0.00	1.00
		
			2010	0.14	0.35	0.00	1.00
		
			2015	0.09	0.30	0.00	1.00
		
			2022	0.08	0.27	0.00	1.00
		
Azores			2005	0.29	0.29	0.00	1.00
		
			2010	0.08	0.28	0.00	1.00
		
			2015	0.13	0.35	0.00	1.00
		
			2022	0.14	0.35	0.00	1.00
		
Madeira			2005	0.34	0.13	0.00	1.00
		
			2010	0.14	0.31	0.00	1.00
		
			2015	0.11	0.31	0.00	1.00
		
			2022	0.09	0.29	0.00	1.00
		
Alentejo			2005	0.08	0.27	0.00	1.00
		
			2010	0.07	0.26	0.00	1.00
		
			2015	0.07	0.26	0.00	1.00
		
			2022	0.07	0.26	0.00	1.00
		
Employed ratio			2005	0.38	0.34	0.00	1.00
		
			2010	0.36	0.35	0.00	1.00
		
			2015	0.37	0.35	0.00	1.00
		
			2022	0.40	0.27	0.00	1.00
		
Unemployed ratio			2005	0.05	0.14	0.00	1.00
		
			2010	0.07	0.17	0.00	1.00
		
			2015	0.08	0.20	0.00	1.00
		
			2022	0.05	0.15	0.00	1.00
		
Retired			2005	0.33	0.42	0.00	1.00
		
			2010	0.33	0.41	0.00	1.00
		
			2015	0.30	0.29	0.00	1.00
		
			2022	0.33	0.43	0.00	1.00
		
Incapacity to work			2005	0.07	0.17	0.00	1.00
		
			2010	0.03	0.13	0.00	1.00
		
			2015	0.14	0.22	0.00	1.00
		
			2022	0.04	0.14	0.00	1.00
		

*In 2022, data only exists for 6th year and 9th year combined

### 3.2 Methods

The empirical analysis in this study follows the World Health Organization (WHO) methodology for measuring financial protection, as outlined by Xu et al. [29] and later revised by the WHO Regional Office for Europe [32]. Catastrophic Health Expenditure (CHE) occurs when a household’s out-of-pocket (OOP) healthcare payments meet or exceed a specified threshold of their capacity to pay [7]. Capacity to pay is calculated as total household expenditure minus subsistence spending [32]. By deducting essential expenditures, the calculation focuses on the portion of the budget remaining after basic needs have been met, representing the resources realistically available for healthcare spending [27]. This makes the concept of capacity to pay a more precise and equitable measure of financial burden compared to methods like budget share, which assume the entire household budget is available for healthcare expenses without accounting for basic needs [31].

In this study, subsistence spending is estimated as the average combined spending on food, rent, and utilities per equivalent person for households whose total consumption per equivalent person lies between the 25th and 35th percentiles of the distribution [32]. This serves as the basis for defining the basic needs level^3^ [32]. The selection of the 25th to 35th percentile range is intended to exclude households that may under-spend on basic needs due to financial constraints while also avoiding those closer to the median, thereby ensuring a more reliable estimate of basic expenditures [27]. This range directly influences the estimation of subsistence spending and, consequently, the catastrophic health expenditure (CHE) reference line. Adjusting the percentile range alters the CHE threshold, with higher ranges leading to an increased CHE measure. Alternative calculations using the 35th to 45th percentile and the 45th to 55th percentile further illustrate how different reference points affect the estimation of basic needs expenditures and, by extension, the CHE assessment. The OECD equivalence scale^4^ is applied to adjust spending for household size and composition [32].

Previous studies have employed various approaches to defining and estimating basic needs expenditures, reflecting the ongoing uncertainty and debate regarding what constitutes “basic needs [27], [40]. Food spending is often used as a proxy for essential expenditures, with different methodologies available for its incorporation [30]. Some studies, including those by the Pan American Health Organization and the World Bank, have based their analysis on actual household food spending [41], [42]. This approach presents limitations, as it does not account for discretionary food spending and further, fails to consider the impact of out-of-pocket healthcare expenditures on household food spending [40]. For instance, a household reducing food expenditure due to high healthcare costs may appear to have greater financial capacity, thereby being less likely to be classified as experiencing catastrophic health expenditure (CHE) compared to a household with higher food spending [27].

An alternative approach, the partial normative food spending method, addresses some of these limitations by deducting a standardized amount of food expenditure from each household’s total spending rather than relying on actual food spending [27]. For households that spend less on food than the standard amount, the method deducts their actual food spending instead, which is why the method is considered only partially normative [27]. A key advantage of this approach is that it sets a threshold that rises with total household expenditure [27]. This ensures that financial hardship is measured more equitably, as it prevents overestimating the ability to pay for lower-income households, who generally allocate a larger portion of their income to basic needs compared to wealthier households [27]. However, Cylus, Thomson, and Evetovits [27] found that despite their conceptual differences, both food-based methods yield similar results in terms of CHE incidence. In the countries analysed, most households report actual food spending below the standardized threshold, resulting in identical CHE classification under both methods [27]. As a result, effective CHE thresholds remain largely unchanged, particularly for poorer households, with many of the same households classified as experiencing catastrophic spending under both approaches [27]. Consequently, this may overstate the ability to pay of poorer households relative to wealthier ones [27].

However, the question remains whether food alone provides an adequate measure of basic needs [43]. The method used in this study builds upon both the actual and normative food-spending methods, but it extends the concept of basic needs by including housing and utilities, such as water, gas, electricity, and heating, thereby aiming to better reflect essential spending [32]. This approach acknowledges that, particularly in high- and middle-income countries, where food spending constitutes a smaller share of overall expenses, the threshold should be dynamic and responsive to income differences [43]. Furthermore, in colder climates, utility expenses such as heating often represent a larger portion of essential spending, reinforcing the need to consider additional expenditures beyond food [43].

Moreover, this framework uses total expenditure to measure capacity to pay, meaning that consumption is the chosen metric in this context. Assessing capacity to pay is possible in two ways: by using income or by using consumption [20]. Measuring capacity to pay based on income assumes households have no access to other resources for healthcare, such as savings or borrowing, which is not entirely accurate [27]. Measuring total consumption, on the other hand, includes health expenditures financed through savings or borrowing, treating these as extensions of a household’s spending capacity rather than limitations [20]. While neither income nor consumption fully captures a household's resources [44], consumption is often preferred in research for two main reasons: it is considered a more reliable measure of welfare, especially in low-income contexts, and it tends to be typically measured with greater precision [27].

Further, a household’s capacity to pay is permitted to be negative if its total expenditure falls below the basic needs level [32]. This adjustment ensures the analysis captures not only households exceeding the specified threshold of their capacity to pay but also those impoverished or further impoverished by out-of-pocket (OOP) healthcare payments [32]. The threshold for Catastrophic Health Expenditure (CHE) is set at 40% of a household's capacity to pay [32]. Additional calculations were performed for thresholds of 30%, 20%, and 10% to provide a more comprehensive analysis. Higher thresholds indicate a greater financial burden before expenditure is considered catastrophic and reduces the chance of classifying discretionary healthcare spending as such, while lower thresholds capture a broader range of cases but may inflate results [40]. The 40% threshold is commonly used as it marks the point at which healthcare costs begin to compromise other essential expenses.

Formally, a household i is said to incur CHE if $\frac{Y_{i}}{\left[x_{i}-f\left(x_{i}\right)\right]}\geq z\;\;if\;\;Y_{i}>0\;\left(1\right)\;$ where $Y_{i}$ = OOP, x = total household expenditure, $f\left(x_{i}\right)$ = subsistence spending and z = threshold [32]. A household *i* would be considered impoverished if $\frac{Y_{i}}{\left[x_{i}-f\left(x_{i}\right)\right]}>1\;if\;Y_{i}>0\;\left(2\right)$ or further impoverished if $\frac{Y_{i}}{\left[x_{i}-f\left(x_{i}\right)\right]}<0$ if $Y_{i}>0\;\left(3\right)$.

To assess the incidence of Catastrophic Health Expenditures (CHE), a headcount was conducted. The headcount is then defined as the proportion of these households within the overall sample: $\frac{1}{N}\sum_{i=1}^{N}E_{i\;}\left(4\right)$ where N represents the sample size, *E*_i_ = 1 if OOP health expenditures as a fraction of capacity to pay meets or exceeds the threshold and *E*_i_ = 0 if OOP health expenditures do not meet or exceed the threshold [45].

### 3.3 The model

The dependent variable, representing the presence of catastrophic health expenditures (CHE), is binary: y = 1 if the household incurs CHE and y = 0 if it does not. Given the binary nature of the outcome, a logistic regression model was employed, as it is well-suited for estimating the probability of an event (y = 0 or y = 1) based on a set of independent variables [46]. The logistic regression model estimates the probability p of a household incurring CHE as follows: Probability of outcome ($\hat{Y_{i}}$) = $\frac{e^{\beta_{0}+\beta_{1}X_{1}+\beta_{2}X_{2}+...\beta_{1}X_{2}}}{1+e^{\beta_{0+\beta_{1}X_{1}+\beta_{2}X_{2}+...+\beta_{i}X_{i}}}}$ (5) [47]. The logistic function ensures that the predicted probabilities lie within the range [0,1] [47]. With reference to previous studies, independent variables covering gender, education, size of household, urbanicity and the number of seniors and juniors in the household were adopted as independent variables [6], [34], [48], [49]. Regional dummy variables were also incorporated to capture differences across regions [6], and various employment status categories (e.g., employed, unemployed, retired, unable to work) were included to account for differences related to work status. The results are presented as odds ratios, which provide a comparative measure of how likely an event is to occur in one group relative to another [46]. The odds ratio represents the relative change in the odds of the outcome associated with a one-unit increase in the predictor variable [46]. In addition to odds ratios, the change in predicted probability as the variable moves from its minimum to maximum value is presented, and marginal effects are also reported to facilitate the interpretation of the magnitude of these relationships. Marginal effects in logit models measure the absolute change in the predicted probability of an outcome occurring when an explanatory variable increases by one unit, holding other variables constant [50]. Since these effects are observation-specific and depend on the values of other covariates, average marginal effects (AMEs) are calculated to avoid the need to choose a single reference point.

## 4 Results

The incidence of CHE declined across all thresholds throughout the study period, reflecting a positive trend (see Table 3). This decline was consistent at higher thresholds (CHE30 and CHE40), while trends at lower thresholds (CHE10 and CHE20) were less steady. At the 10% threshold (CHE10), the incidence increased significantly from 36.10% in 2005 to 43.66% in 2015 before decreasing to 32.70% by 2022. However, this may also be attributed to the stringency of the threshold, supporting the interpretation of a broader positive trend. In fact, this pattern aligns with global findings; the 2023 Global Monitoring Report reported a continuous rise in the proportion of the global population experiencing catastrophic health spending at the 10% threshold from 2000 to 2019^5^ [7]. The rise in catastrophic health spending is consistent with evidence that households are dedicating an increasing share of their rising consumption expenditures to out-of-pocket (OOP) health payments [7]. Globally, the observed increase in catastrophic health spending during the 2000–2019 period occurred alongside a rise in private consumption levels, suggesting that higher consumption did not necessarily improve financial protection in healthcare but instead contributed to greater OOP burdens [7].

**Table 3:** Results for incidence at different thresholds (e.g. CHE40 corresponding to a threshold of 40% of capacity to pay) and impoverishing expenditure in 2005, 2010, 2015 and 2022.

	2005	2010	2015	2022	Δ 2010–2005	Δ 2015–2010	Δ 2022–2015
CHE40	9.42%	8.91%	7.19%	5.04%	−0.51%	−1.72%	−2.15%
CHE30	13.88%	13.26%	11.99%	8.02%	−0.61%	−1.28%	−3.97%
CHE20	21.57%	20.88%	21.25%	14.60%	−0.69%	0.37%	−6.65%
CHE10	36.10%	36.61%	43.66%	32.70%	0.51%	7.04%	−10.95%
Impoverished households (%)	2.40%	3.50%	2.82%	3.42%	1.10%	−0.68%	0.60%

Policy measures may have contributed to improved financial protection in Portugal. Historically, most healthcare services, such as emergency care, GP visits, and specialist consultations, involved flat-rate user charges and co-payments [51]. Although exemptions existed, these were expanded in 2016 to cover approximately 6.1 million NHS users (approx. 60% of the population) [51]. Further, in 2020, fees for primary care and NHS-prescribed services were discontinued [15]. By 2022, all NHS charges were eliminated, except for non-referred emergency care^6^ [15]. At the same time, it must be noted that the pandemic disrupted healthcare access, particularly non-COVID-19 services provided by private healthcare providers, and influenced changes in patients’ healthcare-seeking behaviours [15]. In the five years leading up to the pandemic, private health expenditure in Portugal increased at an average rate of 4% annually, driven largely by higher out-of-pocket (OOP) costs [15]. However, this trend was temporarily disrupted by the pandemic, resulting in a nearly 7% reduction in private health spending in 2020 [15].

**Fig. 1:**
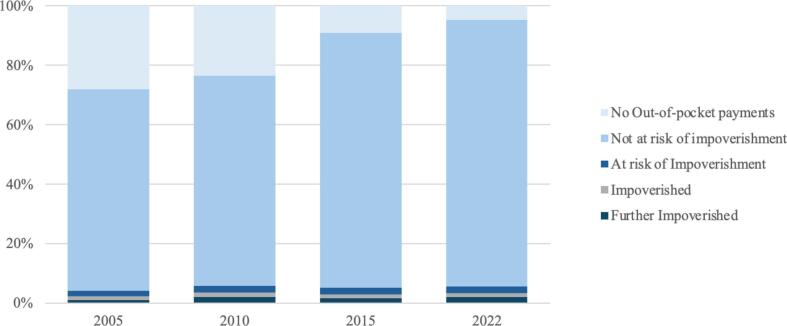
Breakdown of the population by risk of impoverishment after paying out of pocket for health services.

Fig. 1 further illustrates a positive trend, with the proportion of households not at risk of impoverishment from OOP payments rising from 67.70% in 2005 to 89.49% in 2022. Here, “at risk” is defined following the WHO guidelines, where households are considered at risk of impoverishment if their expenditures, after out-of-pocket payments, bring them to within 120% of the basic needs threshold [43]. The figure also shows that the proportion of households with no OOP payments declined significantly, from 28.06% in 2005 to 4.84% in 2022. These two categories, *households not at risk* and *those with no OOP payments* represent the most significant changes over the period. This shift suggests a transition from households previously reporting no OOP payments to those now incurring OOP payments without experiencing financial hardship, indicating that either more households are accessing and utilizing more healthcare services or previously fully health-insurance-covered care has been replaced by services requiring some payment value. As the Portuguese NHS has reduced user charges over the period, this change is more likely to reflect general barriers to access such as waiting times or unavailability within the Portuguese NHS. As a result, more people resort to also using private sector health services and paying out-of-pocket. Barriers to accessing care remain. By 2022, approximately 3% of the Portuguese population reported unmet medical needs, exceeding both the EU average of 2.2% and Portugal's pre-pandemic level of 1.7% [15]. In Portugal, cost remains a leading factor behind unmet healthcare needs^7^ [15].

The stability in the proportions of households classified as “Impoverished,” “At risk of impoverishment,” or “Further impoverished” suggests that while progress has been made, certain vulnerable groups remain at risk. Regarding impoverishing effects of OOP payments, a generally steady pattern with slight fluctuations over time is observable (see Fig. 1). The proportion of households rose from 2.40% in 2005 to 3.50% in 2010, followed by a decrease to 2.82% in 2015. This figure rose again slightly to 3.42% in 2022. The data shows a tendency for the proportion to rise following major economic shocks, such as the 2008 financial crisis and the COVID-19 pandemic in 2022. The proportion of households at risk of impoverishment increased slightly, from 1.84% in 2005 to 2.24% in 2022.

The analysis of household OOP healthcare spending as a percentage of their capacity to pay reveals a shift in spending patterns over the study period. The results suggest a movement away from both very low and very high expenditure levels toward a more balanced distribution of healthcare spending. Both the number of households spending 5% of their capacity to pay and those spending 40% or more decreased. Simultaneously, the proportion of households in mid-range expenditure bands (5%–20%) increased over the study period, indicating a concentration of spending in moderate ranges.

Incidences of CHE were calculated using the 40% threshold for NUTS II regions. At the regional level, CHE incidence generally declined between 2005 and 2022, reflecting overall improvement across all regions. However, variations in both the extent and timing of these reductions were observed across regions throughout the study period. At the beginning of the study period, the North had the lowest incidence rates, while Alentejo recorded the highest rates. By the end of the study period in 2022, Lisbon emerged as the region least affected, whereas Azores was the most affected.

Lisbon and the North consistently showed lower CHE incidence rates compared to other regions. The Centre, Lisbon, and Alentejo achieved reductions of more than half in their CHE incidence rates, with steady declines throughout the study period, reflecting consistent improvements. Alentejo, which had the highest incidence rates at the beginning of the study period in 2005, showed the greatest improvement among all regions by 2022. Despite this progress, Alentejo still recorded higher-than-average incidence rates compared to most other regions at the end of the study period.

In contrast to other regions, the Azores showed an inconsistent trend throughout the study period and recorded the smallest reduction in CHE incidence rates. The region also recorded the highest burden among all regions in 2022. Data show that rural regions such as Alentejo, Madeira, and the Azores face limited healthcare infrastructure, including shortages of specialists and facilities [51]. In addition to these infrastructure challenges, lower income per capita may contribute to the higher CHE incidence rates in these areas. For instance, in 2021, the Azores had a GDP per capita of €18,364, approximately 41.9% lower than Lisbon’s €31,618 [52]. Comparable disparities were observed in Alentejo (€19,709) and Madeira (€20,108), each recording GDP per capita figures more than 35% below that of Lisbon [52]. In lower-income regions, even moderate out-of-pocket healthcare expenditures may represent a substantial share of household expenditure, increasing the likelihood of catastrophic health spending. Given the consistently higher CHE rates observed in these regions and the limited healthcare infrastructure, these challenges point to the need for targeted measures to improve healthcare access and reduce financial strain in affected regions.

Differences are also evident in the number of households experiencing CHE across consumption quintiles (Table 4). The incidence is highest among households in the poorest consumption quintile (1st) and consistently declines as consumption increases, with lower levels observed in higher-consumption quintiles throughout the study period. This pattern is a common finding in studies on catastrophic health expenditures [7], [28], [53], [54], [55]. Over the entire period from 2005 to 2022, there was a net decrease in CHE incidence across all consumption quintiles, though the magnitude of this reduction varied significantly. During 2005-2010, the trend was somewhat mixed. While the 1st and 2nd quintiles saw slight increases in CHE incidence, the 3rd, 4th, and 5th quintiles experienced marginal decreases. In particular, the period between 2010 and 2015 shows divergent trends across consumption quintiles. The 1st quintile saw a significant increase in CHE (3.24 percentage points), reaching its peak incidence in 2015. In contrast, all other quintiles experienced substantial decreases in CHE incidence during this period, with the 2nd quintile seeing the sharpest drop (-7.05 percentage points).

**Table 4:** Incidence in CHE for consumption vs. in income quintiles in 2005, 2010, 2015 and 2022 using 40% threshold.

Consumption								
	2005	2010	2015	2022	2010–2005	2015–2010	2022–2015	Total
1st	18.37%	19.49%	22.73%	18.17%	1.12%	3.24%	−4.55%	−0.20%
2nd	12.88%	14.48%	7.44%	3.64%	1.61%	−7.05%	−3.80%	−9.24%
3rd	7.82%	7.64%	4.23%	1.85%	−0.18%	−3.40%	−2.38%	−5.97%
4th	5.20%	5.11%	1.49%	0.92%	−0.09%	−3.62%	−0.58%	−4.29%
5th	2.84%	2.50%	0.08%	0.63%	−0.34%	−2.41%	0.55%	−2.21%
Income								
	2005	2010	2015	2022	2010–2005	2015–2010	2022–2015	Total
1st	20.20%	19.60%	17.10%	11.10%	−0.60%	−2.50%	−6.00%	−9.10%
2nd	13.60%	12.70%	10.50%	6.30%	−0.90%	−2.20%	−4.20%	−7.30%
3rd	7.10%	5.70%	4.40%	4.70%	−1.40%	−1.30%	0.30%	−2.40%
4th	4.00%	4.60%	2.90%	2.00%	0.60%	−1.70%	−0.90%	−2.00%
5th	2.10%	2.00%	1.00%	1.10%	−0.10%	−1.00%	0.10%	−1.00%

For a comprehensive assessment of the distributional impact, the incidence of catastrophic health expenditure (CHE) was also calculated across income quintiles (Table 4). A particularly interesting finding emerges when comparing CHE incidence across income quintiles versus consumption quintiles. While both approaches reveal a similar socioeconomic gradient, with lower quintiles bearing a higher burden, they diverge significantly in their temporal patterns and the magnitude of changes observed across different periods. The consumption-based measure shows more pronounced fluctuations and particularly suggests a widening gap between the poorest and other quintiles during 2010-2015, whereas the income-based measure indicates more consistent improvements across all income groups.

The divergence in results between income- and consumption-based CHE measures reflects fundamental differences in how household economic capacity is conceptualized. When using consumption as a reference point, the key difference lies in the fact that consumption excludes savings from the denominator. Households may draw on accumulated savings to cover health costs, preventing a drastic fall in current consumption and potentially avoiding the CHE threshold. However, for lower-income households, consumption is closely tied to immediate needs and leaves little room for discretionary spending. These households face several constraints, such as minimal or no savings to draw upon, limited access to credit or informal safety nets and greater vulnerability to income shocks. As a result, changes in out-of-pocket health spending may more quickly push lower-income households over the CHE threshold when measured against consumption, explaining the heightened sensitivity observed in consumption-based measures for the poorest quintiles.

When breaking down out-of-pocket payments by health service, the PHBS facilitates their categorization into six broad groups. Pharmaceuticals consistently remain the largest category of healthcare spending. A study conducted by da Costa et al. in 2016 [56] examining the effects of the economic recession on patients, found that while many patients managed rising medication costs by choosing generics, about 15% resorted to discontinuing medication or extending interdose intervals to reduce expenses. In recognition of the financial burden posed by pharmaceuticals, the *abem* *program* was introduced in Portugal in May 2016 to assist vulnerable populations by covering OOP costs for prescribed medicines in community pharmacies. Initial findings suggest the program has contributed to reducing financial barriers associated with medication access [57]. By 2022, the share of pharmaceuticals had decreased, though it remained the largest expenditure category. Medical services, which ranked as the second-largest category in 2005, have seen a gradual decline over time. In contrast, spending on oral care has increased. Therapeutic devices and materials experienced a significant rise, nearly doubling from 9.03% in 2005 to 18.53% in 2022. Outpatient and inpatient services represent smaller portions of total spending.

### 4.1 Sensitivity of impoverishing health expenditure estimates to basic needs reference points

The results demonstrate that the choice of reference point for estimating basic needs spending influences the measured incidence of catastrophic health expenditures (CHE) and the share of the population experiencing impoverishing health expenditures. As the reference group shifts from households in the 25th–35th percentile to those in the 35th–45th and 45th–55th percentiles of the consumption distribution, CHE rates consistently increase across all thresholds. This is because higher reference points imply a higher basic needs threshold, which, in turn, reduces households' remaining capacity to pay.

The largest difference in CHE incidence between the considered reference groups occurred in 2005. The disparity between the 25th–35th percentile and the 45th–55th percentile reference groups in this year was 2.14 percentage points, indicating that the CHE incidence estimated using the 45th–55th percentile as the reference was approximately 1.33 times higher than that estimated using the 25th–35th percentile. In general, a greater discrepancy is observed in the incidences when comparing the 35th to 45th and 45th to 55th percentile reference points, compared to the 25th to 35th and 35th to 45th percentiles. This observation supports the methodology of choosing the 25th to 35th percentile range, as households closer to the median show increased variation in consumption. However, the data also indicates a general convergence in the CHE estimates obtained using different reference points as the period progresses. For instance, the difference in CHE incidence at the 40% threshold between the 25th-35th and 45th-55th percentile ranges decreased from 2.14% in 2005 to 0.27% in 2022, suggesting reduced sensitivity of the final CHE estimates to the specific choice of reference point in later years.

The effect is more pronounced for impoverishing expenditures, which nearly triple between the lowest and highest reference groups, rising from 2.40% to 7.32% in 2005. In 2022, the pattern remained consistent, with impoverishing expenditures in the 45th–55th percentile approximately 1.33 times higher than in the 25th–35th percentile group. Consequently, while the choice of the reference point might become less critical for CHE over time, it remains a significant determinant of the defined poverty line against which impoverishing health expenditures are measured.

### 4.2 Differences across household types

Understanding how household composition influences exposure to catastrophic health expenditure (CHE) is essential for identifying financial vulnerability within the population. This section examines the incidence of CHE across diverse household types, using a categorization framework developed by Peralta et al. [58].

Peralta et al. [58] calculated poverty rates show that households with children tend to experience higher levels of poverty compared to those without. Specifically, the absolute poverty rate was lowest among single-adult households and highest in households with two adults and three or more children [58]. The authors attribute this to the greater need for household resources required to adequately meet the needs of children and adolescents [58]. Results for CHE incidence show that significant health expenditures do not contribute to the poverty issues of those households. Instead, findings suggest a different picture: households without children may experience greater financial hardship due to health expenditures, in particular when older adults are present, as low income and (relatively) high out-of-pocket expenditures hit them. While catastrophic health expenditure is primarily influenced by health-related vulnerabilities, particularly affecting older households, poverty is more closely linked to the overall resource demands of a household, which are significantly higher in families with children.

**Fig. 2:**
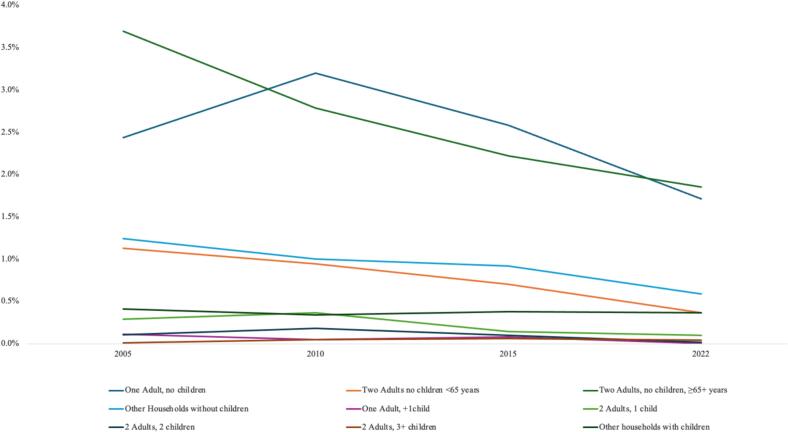
Household composition and absolute CHE incidence.

Over the period from 2005 to 2022, absolute CHE incidence declined across nearly all household types (Fig. 2). The only exception to this downward trend was found in households with two adults and three or more children, where the absolute CHE incidence increased slightly by 0.03 percentage points. Throughout the study period, households without children consistently experienced higher absolute CHE incidence compared to those with children. The highest CHE incidence was observed in single-adult households and in households comprising two adults without children, where at least one adult was aged 65 years or older. In the latter group, the gap between relative and absolute CHE incidence was especially large, with relative rates reaching up to six times the level of absolute rates. This reflects the particular vulnerability of older households and signals the need for targeted measures to ease their financial burden from health-related costs.

### 4.3 Populations characteristics associated with CHE

Table 5 provides detailed insights into the changes in predicted probabilities when variables shift from their minimum to maximum values, along with the corresponding Average Marginal Effects (AME).^8^ Table 6 presents the findings from the multivariate logistic regression analysis.

**Table 5:** Predicted probability changes from minimum to maximum values and average marginal effects (AME).

Characteristics				Min-Max	AME
Consumption			2005	−0.07	−0.01***
			2010	−0.20	−0.01***
		
			2015	−0.26	−0.03***
		
			2022	0.15	−0.02***
		
Household head gender			2005	0.00	0.01
		
			2010	0.04	0.02**
		
			2015	0.03	0.00*
		
			2022	−0.03	0.01**
		
Household head age			2005	0.24	0.00***
		
			2010	0.09	0.00
		
			2015	0.21	0.01**
		
			2022	−0.23	0.00**
		
Household Head completed education					
4th year			2005	−0.01	−0.01
			2010	−0.05	−0.03***
		
			2015	0.01	0.00
		
			2022	0.00	0.00
		
6th year			2005	−0.02	−0.04**
		
			2010	−0.07	−0.05***
		
			2015	−0.02	−0.01
		
			2022	0.00	−0.02**
		
9th year*			2005	−0.03	−0.05***
		
			2010	−0.09	−0.06***
		
			2015	−0.02	−0.01
		
			2022	N/A	N/A
		
12th year (Secondary and post-secondary)			2005	−0.04	−0.10***
			2010	−0.09	−0.06***
		
			2015	−0.09	−0.03**
		
			2022	0.08	−0.02**
		
Higher education			2005	−0.05	−0.11***
		
			2010	−0.10	−0.07***
		
			2015	−0.09	−0.03**
		
			2022	0.00	−0.05***
		
Household Size			2005	0.01	0.00
		
			2010	−0.04	0.00
		
			2015	−0.12	0.00
		
			2022	0.06	0.00
		
Senior Ratio			2005	−0.03	0.02*
		
			2010	0.09	0.04**
		
			2015	0.14	0.04***
		
			2022	−0.07	0.03**
		
Junior Ratio			2005	0.02	−0.07**
		
			2010	−0.08	−0.07**
		
			2015	−0.12	−0.06**
		
			2022	−0.07	−0.04
		
Urban			2005		
			
			2010	Omitted categorical variable	
	
			2015		
	
			2022		
			
Semi-urban			2005	0.00	0.01
		
			2010	0.02	0.01
		
			2015	0.01	0.00
		
			2022	0.03	−0.01**
		
Rural			2005	0.01	0.01
		
			2010	0.02	0.01
		
			2015	−0.01	0.00
		
			2022	0.02	−0.01
		
North			2005	−0.02	−0.03**
		
			2010	0.01	0.01
		
			2015	0.04	0.01
		
			2022	0.04	−0.02**
		
Center			2005	−0.01	−0.01
		
			2010	0.00	0.00
		
			2015	0.06	0.02*
		
			2022	0.02	−0.01
		
Lisboa			2005	−0.01	−0.02
		
			2010	−0.03	−0.02
		
			2015	0.05	0.02
		
			2022	0.02	−0.01
		
Algarve			2005	−0.02	−0.03**
		
			2010	−0.01	−0.01
		
			2015	0.02	0.01
		
			2022	0.01	−0.01
		
Azores			2005	0.00	0.00
		
			2010	0.06	0.03**
		
			2015	0.18	0.05***
		
			2022	−0.08	0.03***
		
Madeira			2005	0.00	0.00
		
			2010	0.06	0.03**
		
			2015	0.16	0.05***
		
			2022	−0.01	0.00
		
Alentejo			2005		
		
			2010	Omitted categorical variable	
		
			2015		
		
			2022		
		
Employed ratio			2005	−0.04	−0.10***
		
			2010	−0.14	−0.11***
		
			2015	−0.16	−0.07***
		
			2022	0.09	−0.05***
		
Unemployed ratio			2005	−0.02	−0.03
		
			2010	−0.04	−0.03
		
			2015	−0.11	−0.04**
		
			2022	0.04	−0.02
		
Retired			2005	0.01	0.01
		
			2010	−0.02	−0.01
		
			2015	−0.01	0.00
		
			2022	−0.01	0.00
		
Incapacity to work			2005	0.01	0.02
		
			2010	−0.01	−0.01
		
			2015	0.00	0.00
		
			2022	−0.12	0.04**
		
***p<0.01	**p<0.05	* p<0.10			

*In 2022, data only exists for 6th year and 9th year combined.

**Table 6:** Odds ratios from logistic regression to assess CHE determinants for Portugal (2005–2022).

Characteristics				Odds ratio	Coefficients
Consumption			2005	0.94***	−0.07***
			2010	0.90***	−0.11***
		
			2015	0.66***	−0.41***
		
			2022	0.74***	−0.30***
		
Household head gender			2005	1.02	0.02
		
			2010	1.24**	0.22**
		
			2015	1.14*	0.14*
		
			2022	1.22**	0.20**
		
Household head age			2005	1.15***	0.14***
		
			2010	1.04	0.04
		
			2015	1.06**	0.06**
		
			2022	1.08**	0.08**
		
Household Head completed education			2005	0.90	−0.10
4th year					
			2010	0.69***	−0.37***
		
			2015	1.03	0.03
		
			2022	1.00	0.00
		
6th year			2005	0.65**	−0.43**
		
			2010	0.57***	−0.56***
		
			2015	0.89	−0.12
		
			2020	0.70**	−0.36**
		
9th year*			2005	0.56***	−0.58***
		
			2010	0.51***	−0.68***
		
			2015	0.90	−0.10
		
			2022	N/A	N/A
		
12th year			2005	0.35***	−1.04***
(Secondary and post-secondary)					
			2010	0.51***	−0.67***
		
			2015	0.64**	−0.45**
		
			2022	0.66**	−0.42**
		
Higher education			2005	0.31***	−1.17***
		
			2010	0.45***	−0.80***
		
			2015	0.63**	−0.46**
		
			2022	0.39***	−0.95***
		
Household Size			2005	1.01	0.01
		
			2010	0.97	−0.03
		
			2015	0.95	−0.05
		
			2022	1.05	0.05
		
Senior Ratio			2005	1.31*	0.27*
		
			2010	1.63**	0.49**
		
			2015	1.80***	0.59***
		
			2022	1.64**	0.49**
		
Junior Ratio			2005	0.45**	−0.80**
		
			2010	0.44*	−0.83*
		
			2015	0.42**	−0.87**
		
			2022	0.50	−0.69
		
Urban			2005		
				
			2010		
				Omitted categorical variable	
			2015		
				
			2022		
				
Semi-urban			2005	1.07	0.07
		
			2010	1.10	0.10
		
			2015	1.03	0.03
		
			2022	0.76**	−0.28**
		
Rural			2005	1.14	0.13
		
			2010	1.10	0.09
		
			2015	0.95	−0.05
		
			2022	0.86	−0.16
		
North			2005	0.69**	−0.37**
		
			2010	1.09	0.08
		
			2015	1.20	0.18
		
			2022	0.71**	−0.35**
		
Center			2005	0.89	−0.12
		
			2010	0.99	−0.01
		
			2015	1.33**	0.29**
		
			2022	0.81	−0.21
		
Lisboa			2005	0.83	−0.19
		
			2010	0.81	−0.21
		
			2015	1.27	0.24
		
			2022	0.85	−0.16
		
Algarve			2005	0.74**	−0.31**
		
			2010	0.92	−0.08
		
			2015	1.08	0.07
		
			2022	0.90	−0.10
		
Azores			2005	0.95	−0.05
		
			2010	1.43**	0.36**
		
			2015	2.16***	0.77***
		
			2022	1.67***	0.51***
		
Madeira			2005	1.01	0.01
		
			2010	1.43**	0.36**
		
			2015	1.97***	0.68***
		
			2022	1.08	0.07
		
Alentejo			2005		
				
			2010	Omitted categorical variable	
			2015		
				
			2022		
				
Employed ratio			2005	0.33***	−1.12***
		
			2010	0.27***	−1.32***
		
			2015	0.37***	−0.99***
		
			2022	0.38***	−0.97***
		
Unemployed ratio			2005	0.70	−0.36
		
			2010	0.74	−0.30
		
			2015	0.56**	−0.58**
		
			2022	0.69	−0.37
		
Retired			2005	1.10	0.09
		
			2010	0.85	−0.16
		
			2015	0.97	−0.03
		
			2022	1.05	0.05
		
Incapacity to work			2005	1.20	0.18
		
			2010	0.92	−0.08
		
			2015	0.99	−0.01
		
			2022	2.14**	0.76**
		
***p<0.01	**p<0.05	* p<0.10			

*In 2022, data only exists for 6th year and 9th year combined.

Equivalized Consumption, measured in increments of 1000€, consistently shows a statistically significant association with catastrophic health expenditure across all years (2005–2022).

While higher consumption seems to naturally lower the likelihood of CHE, the impact remains relatively modest when considering the Average Marginal Effect (AME). The AME for consumption remains close to zero, with a small negative value of -0.01 in 2005 and 2010. This effect slightly intensified over time. This limited effect is reflected by the odds ratios. Despite the statistical significance throughout the study period, the odds ratio for consumption remained relatively close to one, especially in the earlier years, further reinforcing the small economic impact of consumption on CHE across consumption levels.

Education has a significant effect on changes in the Average Marginal Effect (AME) of experiencing catastrophic health expenditures (CHE), with higher levels of education showing consistently statistically significant associations. Households headed by individuals with higher education levels show larger reductions in the AME of experiencing CHE, a pattern that is evident across all years. While lower levels of education, such as completion of the 6th or 9th year, are also associated with a protective effect against CHE, the magnitude of the effect is smaller than that observed for higher education levels and does not always reach statistical significance. Over time, the data demonstrate a clear gradient, highlighting education as a protective factor through its negative association with the likelihood of incurring CHE. This finding is notable, as education and income are generally well established to have a high positive correlation [59], [60].

Household composition reveals both risks and protective factors associated with the likelihood of experiencing catastrophic health expenditures (CHE). The results indicate that age composition shapes CHE risk. Older age emerges as a vulnerability factor, with the senior ratio exhibiting a consistently positive and statistically significant AME, indicating an increased probability of incurring CHE as the proportion of elderly household members rises. In contrast, the junior ratio shows a negative AME, suggesting a protective association between a higher proportion of children in the household and the likelihood of experiencing CHE. This negative association is statistically significant in most years, with AME values between −0.04 and −0.07, indicating a substantial reduction in CHE risk as the share of younger household members increases**.**

While the age of the household head is almost consistently statistically significant, the AME estimates indicate a limited effect on the likelihood of experiencing CHE. It should be noted that relying on the age of the household head as a proxy for the average age of the household has limitations, as it presumes that the head’s age reflects the age distribution of all household members, which may not be accurate. Studies, such as those by Alemayehu et al. [61] and Forget et al. [62], show that healthcare costs vary by life stage, peaking in infancy and senior years. Therefore, measures like senior and junior ratios better reflect the household's overall age composition. As an additional robustness check, a logistic regression was estimated using the computed average age of household members as the sole explanatory variable. The results were consistent with the main specification, indicating that higher average household age is associated with a higher likelihood of experiencing CHE. This finding supports the relevance of household age composition beyond the age of the household head alone.

Overall, these findings are particularly relevant in the context of Portugal’s ageing population [63]. Portugal’s ageing population, low fertility rate, and shrinking working-age population are projected to nearly double the old-age dependency ratio, the proportion of retirees to contributors in the pension system, by 2050 [63]. At the same time, future cohorts of retirees may benefit from improved pension adequacy and living conditions, potentially mitigating the financial vulnerability associated with older age.

The regional results are broadly consistent with the patterns observed in earlier analyses. The North and Algarve show statistically significant negative AMEs in earlier years (−0.03), indicating a lower probability of experiencing CHE relative to Alentejo, the reference region. However, these effects diminish or lose significance over time. In contrast, the autonomous regions show a persistently higher CHE risk. Madeira exhibits positive and statistically significant AMEs in 2010 and 2015, although this association attenuates by 2022. The Azores show a similar but more pronounced pattern, consistently presenting positive and statistically significant AMEs and the highest values compared to other regions, indicating a sustained higher likelihood of CHE in relation to the reference region.

Comparisons of odds ratios between 2005 and 2022 indicate a significant relative improvement in Alentejo over time. Nevertheless, households in Alentejo continue to exhibit a higher likelihood of experiencing CHE compared to most regions. This is consistent with the AME estimates, which show only minimal negative variation, suggesting that Alentejo’s relative position remains less favourable, surpassed only by Madeira and the Azores.

Lastly, changes in the Average Marginal Effect (AME) indicate that employment, as expected, consistently provides a strong protective effect against catastrophic health expenditures (CHE) across all years. In contrast, incapacity to work shows a positive association with CHE risk, becoming statistically significant in 2022, with an AME of 0.04. The AME increases from 0.02 in 2005 to 0.04 in 2022, suggesting a possible strengthening of the relationship over time. However, the estimates do not display a consistent trend across all years. Consequently, households with members unable to work face a higher probability of experiencing CHE, although this pattern should be interpreted with caution given the variability observed across periods.

## 5 Limitations

### 5.1 Stability of results

To assess the stability of the results, computations across all years were conducted using the Partial Normative Food Spending method^9^. The findings remained consistent with those reported in other studies, reinforcing the robustness of the results^10^.

### 5.2 The role of consumption levels

Previous research has shown that higher income or consumption generally reduces the risk of catastrophic health expenditure (CHE) [64], [65], [66]. Accordingly, one result that initially appeared counterintuitive was the rather modest overall impact of consumption on catastrophic health expenditure when analysed across the entire sample. To better understand this pattern, models stratified by quintiles were estimated, revealing a nuanced relationship.

For households in the poorest consumption quintile, higher consumption is associated with a statistically significant positive relationship with CHE in earlier years. In 2005, the Average Marginal Effect (AME) was 0.17, with an odds ratio of 3.37, and this positive association persisted through 2015. These results suggest that, among the poorest households, increases in consumption were linked to a higher probability of experiencing CHE. However, this relationship weakened progressively over time and reversed by 2022, indicating a shift in the association between consumption and CHE for this group and suggesting that the vulnerability of the poorest households may have been mitigated in more recent years.

Several arguments may explain the counterintuitive positive association in earlier periods. Conceptually, one explanation relates to the measurement of household capacity to pay. In this study, total consumption serves as the primary measure of a household’s resources. Unlike income, consumption captures expenditures financed through savings, borrowing, or asset sales, reflecting household coping strategies in response to health shocks. For poor households, medical emergencies often necessitate borrowing or selling assets to cover healthcare costs [9], [67]. In the data, such responses appear as an increase in total consumption during the period of the health shock. In this context, the positive association between consumption and CHE likely reflects endogeneity: higher observed consumption does not lead to CHE, rather, catastrophic health spending itself raises measured consumption. This mechanism is particularly relevant for households with limited discretionary resources, for whom health expenditures constitute a large share of total spending.

To assess the robustness of this interpretation, the analysis was replicated using income-based models, including specifications stratified by income quintiles. Both odds ratios and Average Marginal Effects (AMEs) were estimated for the full sample and for stratified models. These alternative specifications yielded qualitatively similar results, including a positive association for the poorest group in earlier years. This consistency suggests that the observed pattern is not driven by the choice of consumption as the resource measure.

A second explanation concerns the structural differences in health expenditures across consumption quintiles. While catastrophic health expenditure (CHE) among wealthier households is typically driven by high-cost inpatient care or major medical procedures [54], [68], evidence suggests that for the poorest households, CHE is frequently triggered by relatively small, routine expenses, such as outpatient visits or essential medicines [54], [68]. For these households, marginal increases in consumption do not necessarily provide a financial buffer but rather lower the barrier to formal healthcare, converting previously latent or unmet medical needs into realized out-of-pocket expenditures. This pattern is consistent with evidence showing that the income elasticity of health expenditures is highest among low-income and uninsured groups [68], [69]. Small changes in available resources lead to disproportionately large adjustments in spending on urgent healthcare. Since the capacity to pay remains low, even modest health spending is likely to exceed the catastrophic threshold [54], [68], [70]. Consequently, for the lowest quintile, small increases in consumption do not necessarily provide financial protection, but instead reflect limited liquid resources that are quickly exhausted when households face a health shock, increasing the probability of experiencing CHE.

The transition from a positive to a reversed association between consumption and CHE in the lowest quintile must also be evaluated through the lens of institutional risk-pooling. Catastrophic expenditure is often conceptualized as a direct consequence of a failure in financial risk protection [40], [67], [71], [72], [73]. Ideally, risk-pooling mechanisms, such as tax-funded health systems or social insurance that decouple healthcare utilization from a household’s immediate financial capacity [72], [73]. However, the Portuguese context during the study period presents a complex dual reality. On one hand, reduced access to the Portuguese NHS has led to increased reliance on private services and out-of-pocket costs. National-level data indicates a troubling trend: the share of out-of-pocket (OOP) payments as a percentage of total health expenditure has risen steadily, and absolute OOP costs have increased for the general population [16], a level that has historically been high in the European context [15], [16]. Despite this general trend toward privatization and higher OOP burdens, our findings suggest a different path for the most vulnerable. The reversal of the positive association between consumption and CHE by 2022 indicates that the poorest households may have been increasingly shielded from the financial consequences of seeking care. This “mitigation effect” likely stems from targeted policy interventions within the Portuguese system, such as the expansion of user-fee exemptions and the strengthening of social safety nets specifically designed for the most vulnerable segments of the population. Nevertheless, it is critical to emphasize that this statistical reversal does not imply a resolution of financial hardship. CHE remains disproportionately concentrated among lower income and uninsured households. While the “slope” of the relationship has flattened or reversed, indicating a decoupling of marginal gains from immediate risk, the baseline probability of CHE for the poorest households remains significantly higher than that of wealthier cohorts.

In contrast, for the second and third consumption quintiles, higher consumption is consistently associated with a statistically significant reduction in CHE risk across all periods. The negative AME estimates for these lower-middle groups confirm that, once a certain resource threshold is met, additional consumption serves as a genuine financial buffer. Among the fourth and fifth (richest) income quintiles, the impact of consumption on CHE is largely negligible and often not statistically significant. Their AME values are consistently very close to zero, indicating that for wealthier households, variations in consumption have little to no additional effect on their likelihood of experiencing CHE.

## 6 Conclusion

This study provides a comprehensive assessment of the evolution of financial protection against health expenditures in Portugal between 2005 and 2022, revealing clear improvements in financial protection alongside persisting inequalities. Overall, the findings indicate a marked improvement in financial protection over time, with the incidence of catastrophic health expenditure (CHE) declining from 9.4% in 2005 to 5.0% in 2022. This downward trend suggests that policy efforts implemented within the Portuguese health system have contributed to reducing the financial burden associated with healthcare utilization.

Evidence from this analysis points to a growing degree of protection among the poorest households, likely reflecting expanded fee exemptions and strengthened social safety policies. Despite this progress, CHE remains disproportionately concentrated among lower-income and uninsured households, and the baseline probability of financial hardship continues to be significantly higher for poorer groups than for wealthier cohorts.

Access to healthcare has generally increased, with more households utilizing a broader range of healthcare services and fewer people are now at risk of experiencing CHE. However, these developments require cautious interpretation, as reduced access to the Portuguese NHS has led to increased reliance on private services and out-of-pocket costs. This shift toward private provision may undermine the system's equity objectives and signals the need for strategic public investment in healthcare infrastructure and workforce capacity.

Our findings identify specific vulnerable groups requiring targeted policy attention. Elderly households continue to face elevated CHE risk, likely reflecting age-related healthcare needs and insufficient coverage of long-term care services. Households including at least one senior adult show particular vulnerability to CHE. Similarly, households with members unable to work due to disabilities face disproportionate financial burden, suggesting gaps in disability-related healthcare coverage and social protection. Unmet needs and ongoing vulnerabilities remain, particularly among lower-income groups and in disadvantaged regions.

Regional disparities further contribute to unequal financial protection outcomes. Households in the Azores, Madeira, and Alentejo face higher CHE risks, associated with weaker healthcare infrastructure, limited service availability, and persistent financial barriers to care, particularly in regions with lower GDP per capita. Addressing these territorial inequalities will be essential to improving equity across the health system.

In conclusion, although the decline in CHE incidence represents an important achievement, financial protection remains unevenly distributed across socioeconomic groups and regions. Strengthening public healthcare capacity, improving coverage for ageing and disabled populations, and reducing regional disparities will be critical to ensuring equitable access to healthcare and minimizing financial hardship. Sustained and targeted policy action will be necessary to consolidate recent gains and advance Portugal’s progress toward universal health coverage.

**Ethics approval and consent to participate**

Not applicable.

**Consent for publication**

Not applicable.

**Availability of data and materials**

The data used in this study were provided by INE - Statistics Portugal. The usual disclaimers apply. Due to privacy and data protection restrictions, the dataset is not publicly available but may be accessed under specific conditions through INE - Statistics Portugal.

**Funding**

This work used infrastructure and resources funded by Fundação para a Ciência e a Tecnologia (UID/ECO/00124/2013, UID/ECO/00124/2019 and Social Sciences DataLab, Project 22209), POR Lisboa (LISBOA-01-0145-FEDER-007722 and Social Sciences DataLab, Project 22209) and POR Norte (Social Sciences DataLab, Project 22209).

## CRediT authorship contribution statement

**Tzi Kieu Tao:** Writing – review & editing, Writing – original draft, Methodology, Formal analysis, Conceptualization. **Pedro Pita Barros:** Writing – review & editing, Supervision, Methodology, Conceptualization.

## Declaration of competing interest

The authors declare the following financial interests/personal relationships which may be considered as potential competing interests: The data used in this study were provided by INE - Statistics Portugal. The usual disclaimers apply. Due to privacy and data protection restrictions, the dataset is not publicly available but may be accessed under specific conditions through INE - Statistics Portugal. This work used infrastructure and resources funded by Fundação para a Ciência e a Tecnologia (UID/ECO/00124/2013, UID/ECO/00124/2019 and Social Sciences DataLab, Project 22209), POR Lisboa (LISBOA-01-0145-FEDER-007722 and Social Sciences DataLab, Project 22209) and POR Norte (Social Sciences DataLab, Project 22209). If there are other authors, they declare that they have no known competing financial interests or personal relationships that could have appeared to influence the work reported in this paper.

## Figures and Tables

**Table A1:** Incidence of CHE in Portuguese Regions in 2005, 2010, 2015 and 2022 using 40% threshold

	2005	2010	2015	2022	Δ 2010–2005	Δ 2015–2010	Δ 2022–2015	Δ Total
North	8.82%	10.21%	7.94%	4.73%	1.39%	−2.27%	−3.21%	−4.09%
Centro	13.55%	12.17%	10.90%	6.75%	−1.38%	−1.27%	−4.15%	−6.80%
Lisboa	8.96%	6.79%	6.14%	4.20%	−2.17%	−0.65%	−1.94%	−4.76%
Alentejo	16.21%	12.65%	8.28%	7.36%	−3.56%	−4.37%	−0.92%	−8.85%
Algarve	10.51%	8.79%	6.38%	5.32%	−1.72%	−2.41%	−1.06%	−5.19%
Açores	11.48%	14.65%	16.19%	11.22%	3.17%	1.54%	−4.97%	−0.26%
Madeira	13.69%	14.05%	12.98%	7.54%	0.36%	−1.07%	−5.44%	−6.15%
Country	9.42%	8.91%	7.19%	5.04%	−0.51%	−1.72%	−2.15%	−4.38%

**Table B1:** Distribution of out-of-pocket payments (OOP) by health service for whole sample.

	2005	2010	2015	2022
Pharmaceuticals	49.07%	50.79%	53.57%	44.03%
Therapeutic devices & Materials	9.03%	6.84%	6.59%	18.53%
Medical Services	16.29%	14.80%	16.74%	14.97%
Oral care	12.53%	16.54%	12.21%	17.16%
Outpatient Services	8.60%	8.21%	8.13%	0.18%
Inpatient Services	4.47%	2.82%	2.76%	5.07%

**Table C1:** Incidence of catastrophic health expenditure according to different choice of reference points.

25th to 35th Percentile							
	2005	2010	2015	2022	Δ 2010–2005	Δ 2015–2010	Δ 2022–2015
CHE40	9.42%	8.91%	7.19%	5.04%	−0.51%	−1.72%	−2.15%
CHE30	13.88%	13.26%	11.99%	8.02%	−0.61%	−1.28%	−3.97%
CHE20	21.57%	20.88%	21.25%	14.60%	−0.69%	0.37%	−6.65%
CHE10	36.10%	36.61%	43.66%	32.70%	0.51%	7.04%	−10.95%
Impoverished Households	2.40%	3.50%	2.82%	3.42%	1.10%	−0.68%	0.60%
35th to 45th Percentile							
	2005	2010	2015	2022	Δ 2010−2005	Δ 2015−2010	Δ 2022−2015
CHE40	10.40%	9.58%	8.05%	5.29%	−0.82%	−1.53%	−2.76%
CHE30	14.84%	14.01%	13.09%	8.35%	−0.83%	−0.92%	−4.74%
CHE20	22.55%	21.69%	22.67%	15.14%	−0.85%	0.97%	−7.52%
CHE10	36.87%	37.11%	44.64%	33.25%	0.24%	7.53%	−11.39%
Impoverished Households	0.00%	4.39%	3.71%	4.16%	4.39%	−0.68%	0.45%
45th to 55th Percentile							
	2005	2010	2015	2022	Δ 2010−2005	Δ 2015−2010	Δ 2022−2015
CHE40	12.54%	10.29%	8.78%	5.55%	−2.25%	−1.51%	−3.22%
CHE30	17.17%	14.60%	14.13%	8.74%	−2.57%	−0.47%	−5.39%
CHE20	24.99%	22.43%	24.29%	15.78%	−2.55%	1.86%	−8.51%
CHE10	38.12%	37.50%	45.38%	33.89%	−0.61%	7.88%	−11.48%
Impoverished Households	7.32%	5.40%	4.95%	4.69%	−1.92%	−0.45%	−0.26%

**Table D1:** Household composition and poverty rates (absolute and relative).^a^

	**2022**		
	Relative	Absolute	
**Without Children**	14.4%	4.5%	9.9%
One Adult, no children	22.5%	2.1%	20.4%
Two Adults no children <65 years	13.4%	5.7%	7.7%
Two Adults, no children, ≥65+ years	14.4%	2.7%	11.7%
Other Households without children	10.1%	5.9%	4.2%
			0.0%
**With Children**	18.4%	21.2%	−2.8%
One Adult, +1child	29.9%	31.1%	−1.2%
2 Adults, 1 child	11.3%	11.0%	0.3%
2 Adults, 2 children	13.3%	19.5%	−6.2%
2 Adults, 3+ children	25.1%	49.2%	−24.1%
Other households with children	22.6%	40.5%	−17.9%

^a^Absolute poverty was calculated by comparing disposable income to the cost of a defined consumption basket. Following the Orshansky method, individuals were in absolute poverty if their income was less than 3.3 times the cost of this basket. The relative poverty risk threshold is defined as 60 percent of the national median equivalised income [34].

**Table E1:** Household composition and CHE incidence.

	**2005**		**2010**		**2015**		**2022**	
Households	Relative	Absolute	Relative	Absolute	Relative	Absolute	Relative	Absolute
**Households without children**	12.19%	8.50%	10.46%	7.93%	8.83%	6.43%	5.81%	2.66%
One Adult, no children	14.55%	2.43%	14.92%	3.20%	11.83%	2.58%	6.93%	1.72%
Two Adults, no children, <65 years	7.31%	1.13%	5.38%	0.94%	4.18%	0.70%	2.28%	0.37%
Two Adults, no children, ≥65+ years	24.38%	3.69%	17.75%	2.79%	13.63%	2.22%	10.06%	1.85%
Other Households without children	5.54%	1.24%	4.73%	1.00%	5.15%	0.92%	3.16%	0.59%
**Households with children**	3.06%	0.92%	4.08%	0.99%	2.81%	0.76%	2.35%	0.77%
One Adult, +1 child	8.34%	0.11%	3.22%	0.05%	3.51%	0.08%	0.24%	0.00%
2 Adults, 1 child	2.60%	0.29%	4.48%	0.37%	1.41%	0.14%	1.33%	0.10%
2 Adults, 2 children	1.55%	0.11%	4.06%	0.18%	1.92%	0.10%	0.43%	0.01%
2 Adults, 3+ children	0.85%	0.01%	5.30%	0.05%	8.84%	0.06%	2.71%	0.04%
Other households with children	4.12%	0.41%	3.73%	0.34%	4.28%	0.38%	4.18%	0.36%

**Table F1:** Coefficients and odds ratios from multivariate logistic regression models stratified by consumption quintiles to assess consumption variable for Portugal (2005-2022).

		2005				2010				2015				2022				
			Odds ratio		Coefficients		Odds ratio		Coefficients		Odds ratio		Coefficients		Odds ratio		Coefficients	
Logistic Regression Model (multivariate)																		
Income (in 1000€)																		
1. Quintile			3.37		1.21	***	1.81		0.59	***	1.26		0.23	***	0.79		−0.23	***
2. Quintile			0.69		−0.37	**	0.56		−0.58	***	0.58		−0.55	***	0.52		−0.65	***
3. Quintile			0.84		−0.17		0.73		−0.31	**	0.67		−0.40	**	0.73		−0.32	
4. Quintile			0.91		−0.09		0.78		−0.25	*	0.58		−0.54	**	1.30		0.26	
5. Quintile			0.96		−0.04		1.01		0.01		0.93		−0.07		0.95		−0.05	

**Table G1:** Predicted probability changes from minimum to maximum values and average marginal effects (AME) stratified by consumption quintiles to assess consumption variable for Portugal (2005–2022).

			2005			2010			2015			2022	
			Min-Max	AME		Min-Max	AME		Min-Max	AME		Min-Max	AME
Income (in 1000€) (multivariate)													
1. Quintile			−0.26	0.17		−0.17	0.09		−0.05	0.04		0.09	−0.03
2. Quintile			0.02	−0.04		0.11	−0.07		0.04	−0.05		0.05	−0.03
3. Quintile			0.01	−0.02		0.07	−0.02		0.03	−0.02		0.00	−0.01
4. Quintile			0.02	−0.01		0.12	−0.01		0.00	−0.01		0.00	0.00
5. Quintile			0.00	0.00		−0.01	0.00		0.00	0.00		0.00	0.00

**Table H1:** Coefficients and odds ratios from logistic regression to assess CHE determinants for Portugal (2005–2022) based on income.

Characteristics			2005			
			Odds ratio		Coefficients	
Income (in 1000€)			0.95		−0.05	***
Household head gender			0.93		0.07	
Household head age			1.13		0.13	***
Household Head completed education						
4th year			0.90		−0.10	
6th year			0.72		−0.33	**
9th year*			0.63		−0.46	**
12th year (Secondary and post-secondary)			0.36		−1.02	***
Higher education			0.52		−0.65	**
Household Size			1.18		0.16	***
Senior Ratio			1.30		0.26	*
Junior Ratio			0.29		−1.24	**
Semi-urban			1.04		0.04	
Rural			1.11		0.10	
North			0.65		−0.44	***
Center			0.85		−0.16	
Lisbon			0.81		−0.21	
Algarve			0.71		−0.34	**
Azores			0.96		−0.04	
Madeira			1.00		0.00	
Alentejo	(omitted categorical variable)					
Employed ratio			0.43		−0.85	***
Unemployed ratio			0.66		−0.41	
Retired			1.36		0.31	
Incapacity to work			1.34		0.29	
Constant						
N			10403			

Characteristics			2010			
			Odds ratio		Coefficients	
Income (in 1000€)			0.95		−0.05	***
Household head gender			1.21		0.19	**
Household head age			1.01		0.01	**
Household Head completed education						
4th year			0.70		−0.36	***
6th year			0.59		−0.53	***
9th year*			0.56		−0.59	***
12th year (Secondary and post-secondary)			0.58		−0.54	**
Higher education			0.68		−0.38	*
Household Size			1.24		0.22	***
Senior Ratio			1.77		0.57	***
Junior Ratio			0.30		−1.22	**
Semi-urban			1.09		0.08	
Rural			1.09		0.09	
North			1.01		0.01	
Center			0.96		−0.04	
Lisbon			0.82		−0.20	
Algarve			0.91		−0.09	
Azores			1.68		0.52	***
Madeira			1.52		0.42	**
Alentejo	(omitted categorical variable)					
Employed ratio			0.38		−0.96	***
Unemployed ratio			0.81		−0.21	
Retired			0.98		−0.02	
Incapacity to work			1.00		0.00	
Constant					−2.15	
N			9489			

Characteristics			2015			
			Odds ratio		Coefficients	
Income (in 1000€)			0.93		−0.07	***
Household head gender			1.09		0.08	
Household head age			1.06		0.06	**
Household Head completed education						
4th year			0.89		−0.12	
6th year			0.74		−0.30	**
9th year*			0.68		−0.38	**
12th year (Secondary and post-secondary)			0.47		−0.76	***
Higher education			0.45		−0.79	***
Household Size			1.40		0.33	***
Senior Ratio			1.97		0.68	***
Junior Ratio			0.20		−1.59	***
Semi-urban			1.17		0.16	*
Rural			1.10		0.10	
North			1.21		0.19	
Center			1.42		0.35	**
Lisbon			1.27		0.24	*
Algarve			1.00		0.00	
Azores			2.87		1.06	***
Madeira			2.36		0.86	***
Alentejo	(omitted categorical variable)					
Employed ratio			0.47		−0.76	***
Unemployed ratio			0.60		−0.51	**
Retired			1.04		0.04	
Incapacity to work			1.02		0.02	
Constant					−2.84	
N			11398			

Characteristics			2022			
			Odds ratio		Coefficients	
Income (in 1000€)			0.95		−0.05	***
Household head gender			1.19		0.17	**
Household head age			1.10		0.10	***
Household Head completed education						
4th year			0.86		−0.15	
6th year			0.58		−0.54	**
9th year*						
12th year (Secondary and post-secondary)			0.54		−0.61	**
Higher education			0.36		−1.03	***
Household Size			1.56		0.45	***
Senior Ratio			1.93		0.66	**
Junior Ratio			0.20		−1.59	**
Semi-urban			0.76		−0.28	**
Rural			0.85		−0.17	
North			0.75		−0.29	**
Center			0.98		−0.02	
Lisbon			0.99		−0.01	
Algarve			0.92		−0.08	
Azores			2.28		0.82	***
Madeira			1.32		0.28	*
Alentejo	(omitted categorical variable)					
Employed ratio			0.44		−0.82	**
Unemployed ratio			0.74		−0.30	
Retired			1.25		0.22	
Incapacity to work			2.56		0.94	***
Constant					−3.75	
N			11701			

**Fig. A1:**
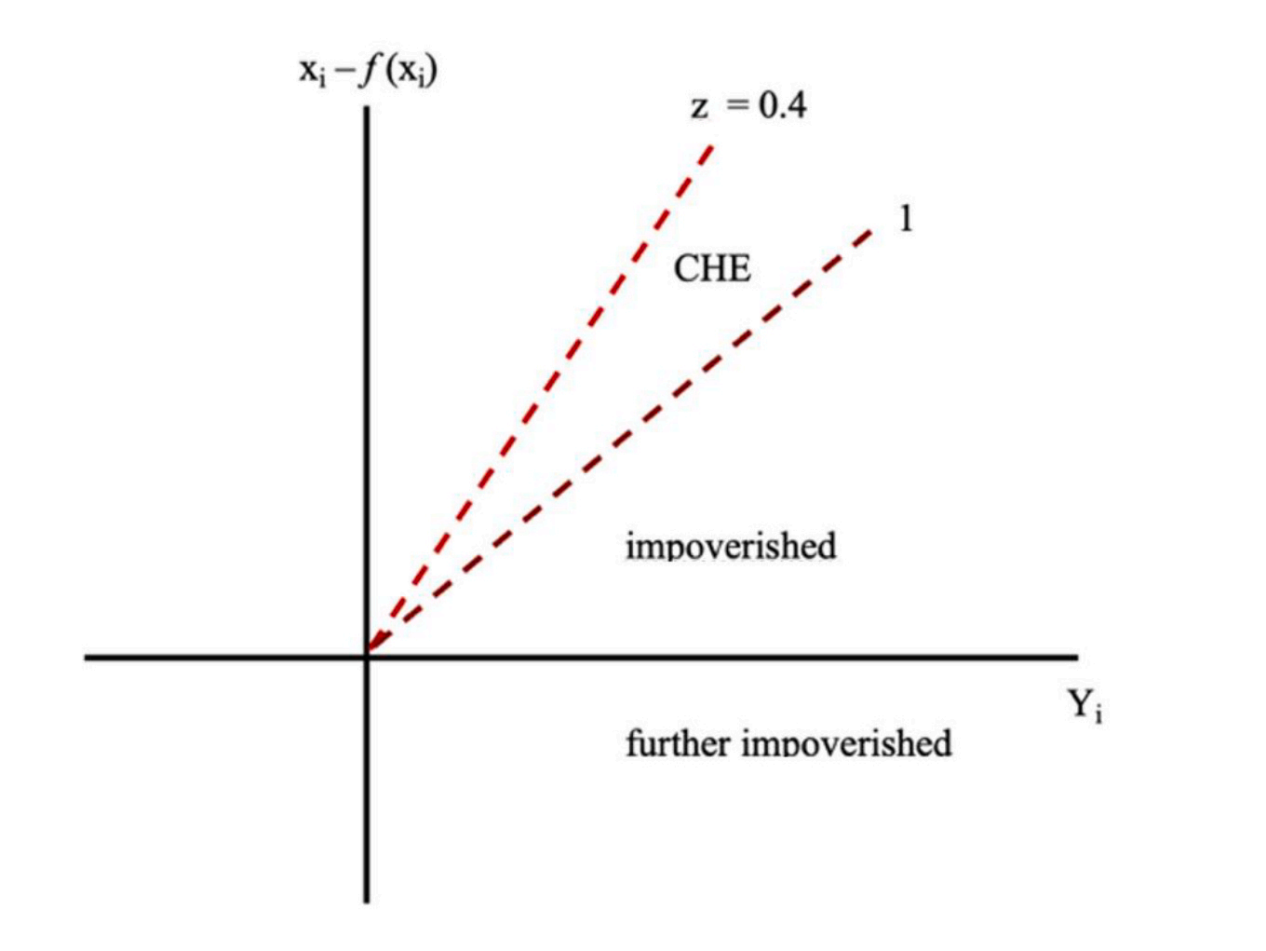
Conceptual representation of CHE, impoverishing and further impoverishing healthcare expenditure.

**Fig. B1:**
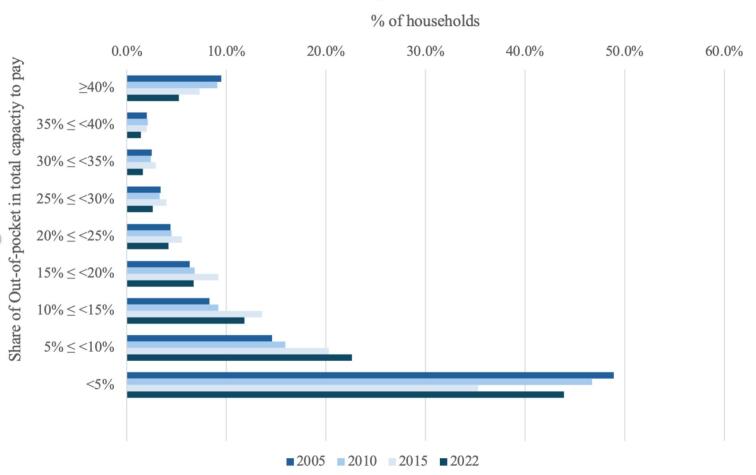
Distribution of households according to their share of Out-of-pocket payments (OOP) in total capacity to pay.

**Fig. C1:**
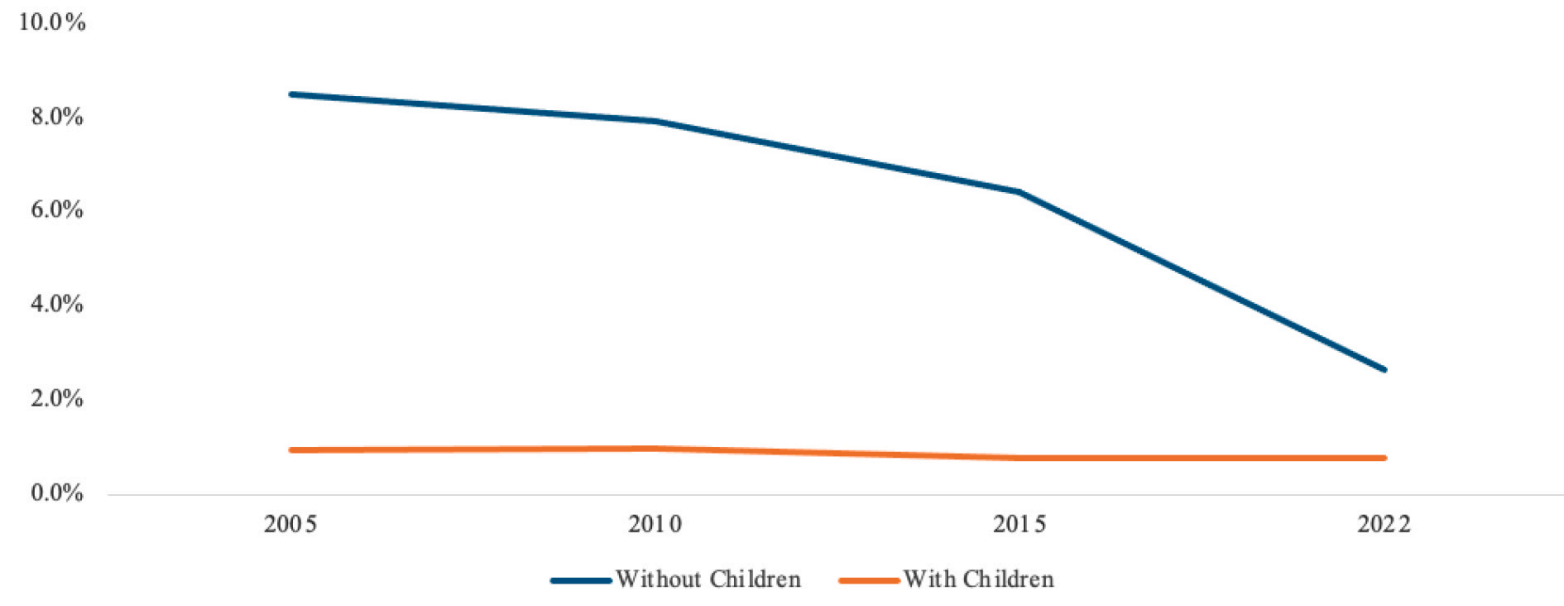
Absolute CHE Incidence comparison of households with and without children.

## Footnotes

1 Unmet needs can arise from factors such as financial constraints, geographic distance to health facilities, and long waiting times [33].

2 The distinction lies between healthcare expenditures that are substantial in absolute terms and those that are significant relative to a patient’s ability to pay [69].

3 Minimum level of expenditure required to meet essential living needs (food, rent, and utilities)

4 Equivalence scales allow for the consideration of economies of scale (e.g., shared costs for rent and utilities) and recognizes that children typically require fewer resources than adults. A single-person household would require relatively more income per person to cover basic needs compared to a family of four, where resources are shared.

5 A different calculation method was used in this study.

6 Unless accessed via NHS phone line (SNS 24), primary care units or other body of the NHS [34]

7 Unmet needs can arise from factors such as financial constraints, geographic distance to health facilities, and long waiting times [32]

8 To validate the findings, multivariate logistic regression and average marginal effects (AME) analyses were also conducted using income as the explanatory variable instead of equivalized consumption. The results demonstrated consistent patterns with the main analysis. These supplementary findings are presented in the appendix.

9 The partial normative food spending method, also used by the WHO, estimates a household’s capacity to pay by subtracting a standard amount for basic food needs from total consumption, rather than accounting for other essential spending like housing, rent, or utilities. Catastrophic health spending is identified when out-of-pocket payments exceed a set share (e.g., 40%) of that capacity.

10 Detailed results are available upon request from the authors.
